# Prevalence of delirium among older nursing home residents: a systematic review and meta-analysis

**DOI:** 10.1007/s41999-026-01422-0

**Published:** 2026-02-19

**Authors:** Alexandre Houdelet-Oertel, Romy Lauer, Vincent Molitor, Roberto Walter, Jonas Dörner, Rebecca Palm, Ina Otte, Horst Christian Vollmar, Bernhard Holle

**Affiliations:** 1 German Center for Neurodegenerative Diseases (DZNE), Stockumer Str. 12, 58453 Witten, Germany; 2 Faculty of Health, School of Nursing Science, Witten/Herdecke University, Alfred-Herrhausen-Str. 45, 58455 Witten, Germany; 3 Faculty of Medicine, Institute of General Practice and Family Medicine (AM RUB), Ruhr University Bochum, Universitätsstr. 150, 44801 Bochum, Germany; 4 Department of Medical Informatics, Biometry and Epidemiology (AMIB), Ruhr University Bochum, Universitätsstr. 150, 44801 Bochum, Germany; 5 School VI - Medicine and Health Sciences, Carl von Ossietzky Universität Oldenburg, Ammerländer Heerstr. 114-118, 26129 Oldenburg, Germany; 6 Faculty of Medicine, Department of Health Services Research, Institute for Diversity Medicine, Ruhr University Bochum, Universitätsstr. 150, 44801 Bochum, Germany

**Keywords:** Delirium, Prevalence, Nursing home, Systematic review, Meta-analysis, Associated factors

## Abstract

**Purpose:**

Delirium is a common geriatric syndrome that presents as an acute change in mental status and is associated with adverse outcomes. The primary aim of this systematic review and meta-analysis was to determine its prevalence in the nursing home population.

**Methods:**

MEDLINE, CINAHL, PsycInfo, and Web of Science were searched up to July 11, 2025. Eligible studies reported cross-sectional prevalence data on delirium in the nursing home population, identified using formal diagnostic criteria (DSM-III or ICD-10 onwards) or validated detection tools. Two reviewers independently screened records, extracted data, and assessed quality. A random-effects meta-analysis was conducted to pool the overall prevalence. The reporting adhered to the Preferred Reporting Items for Systematic Reviews and Meta-Analyses (PRISMA) guidelines.

**Results:**

A total of nine studies were included, and prevalence data on delirium from eight distinct study populations were analyzed. The prevalence of delirium ranged from 1.0% to 57.9%. The pooled prevalence, encompassing 4291 nursing home residents aged ≥ 65 years, was 18.8% (95% CI: 7.4%–40.2%; I2 = 96.9%; Wald-Chi2-Test = 227.66, *p* < 0.001). Dementia was present in up to 86.2% of residents. Four studies reported factors associated with the prevalence of delirium, with cognitive impairment most frequently identified as a factor in all four studies.

**Conclusion:**

Delirium was identified as a common health issue in most of the included studies, although prevalence rates were heterogeneous. Future research should prioritize the development of effective detection and prevention strategies, the validation of tools tailored to the nursing home setting, and the conduct of robust, internationally comparable prevalence assessments.

**Supplementary Information:**

The online version contains supplementary material available at 10.1007/s41999-026-01422-0.

## Key summary points

**Aim:**

This systematic review and meta-analysis primarily aimed to determine the prevalence of delirium in the nursing home population.

**Findings:**

Across eight distinct study populations comprising 4291 nursing home residents aged ≥ 65 years, delirium prevalence ranged from 1.0% to 57.9%, with a pooled estimate of 18.8% (95% CI: 7.4%–40.2%; I2 = 96.9%).

**Message:**

The considerable heterogeneity in delirium prevalence highlights the need for validated detection tools tailored to the nursing home setting, as well as for more robust and internationally comparable prevalence assessments.

**Supplementary Information:**

The online version contains supplementary material available at 10.1007/s41999-026-01422-0.

## Introduction

Delirium is a neurocognitive syndrome characterized by an acute onset of impaired consciousness [1]; it reflects the brain’s direct response to a wide range of underlying physical etiologies, including medical illness, surgery, drug use, or trauma [2]. Clinically, delirium manifests as disturbances in higher-order cognitive functions, with inattention as the hallmark feature [3]. Additional features include disorientation, memory impairment, language disturbances, disruption of the sleep–wake cycle, as well as psychomotor change [3–7]. The symptoms typically fluctuate in severity and represent a deviation from the individual’s baseline status [1].

Delirium is associated with serious consequences, including increased risk of mortality, institutionalization, new dementia, and further cognitive decline [8–10]. In addition, delirium causes considerable distress for affected individuals, their caregivers, and healthcare staff [11]. Despite its impact, delirium is frequently under-recognized, most likely due to the overlap of its symptoms with dementia and the less conspicuous presentation of hypoactive cases [12, 13]. However, early recognition is crucial because timely medical interventions can reduce the severity and duration of episodes [14] and may improve outcomes [15]. A variety of associated factors contribute to the development of delirium. These factors range from predisposing conditions such as older age, dementia, or sensory impairments to acute precipitants, such as neuroleptics or physical restraints, thereby underscoring its multifactorial nature [2].

Given that nursing home residents are usually of advanced age and often affected by cognitive and functional deficits [16–18], they constitute a particularly high-risk population. Nevertheless, the reported prevalence rates of delirium in this population vary widely. A narrative review published in 2022 [19] reported estimates ranging from 1.4% to 70.3%. This variation is likely attributable to the broad scope of that review, which included studies reporting delirium prevalence rates in nursing home residents without distinguishing between unselected nursing home populations and specific resident groups, or between different methodological approaches. No systematic review has focused specifically on this topic within the past decade [20]. As a result, an up-to-date quantitative synthesis employing consistent methodological criteria is needed.

Therefore, the primary aim of this systematic review and meta-analysis was to examine the point prevalence of delirium among the nursing home population. The secondary aim was to identify factors associated with the prevalence of delirium in nursing home residents, as reported in the included studies, to gain a more comprehensive understanding of their relevance.

## Method

This review was registered in the International Prospective Register of Systematic Reviews (PROSPERO) database with the registration number CRD42023442312. Covidence (Veritas Health Innovation, Melbourne, Australia) was utilized to manage a significant portion of the review process, including handling the systematic search results, screening for eligible studies, data extraction, and quality assessment. The review was reported in accordance with the Preferred Reporting Items for Systematic Reviews and Meta-Analyses (PRISMA) guidelines [21].

### Search strategy

The MEDLINE (via PubMed), Web of Science (Core Collection), CINAHL (via EBSCO), and PsycInfo (via EBSCO) electronic databases were systematically searched. The initial search strategy was developed for MEDLINE (via PubMed) and included the following keywords (combined with appropriate Medical Subject Headings [MeSH] when possible) to search titles and abstracts: “delirium” AND “nursing home” AND “prevalence”. The detailed search strategy, including all additional keywords and MeSH terms, is provided in Supplementary Material 1. This strategy was adapted for use in the other databases. The databases were searched up to May 10, 2023, and updated on July 11, 2025, with no restrictions on publication date. To minimize the risk of publication bias, a manual search was conducted. This included searching for grey literature, such as reports not published in indexed peer-reviewed journals. Therefore, the OAIster, Bielefeld Academic Search Engine (BASE), and Open Access Theses and Dissertations (OATD) databases were searched. The reference lists of all included studies and previous reviews on this topic [19, 20] were manually searched to identify relevant publications.

### Eligibility criteria

The eligibility criteria of this review are presented based on the CoCoPop framework (Condition, Context, and Population) [22]. In addition, the types of eligible studies were specified:

#### Condition

Studies were included if they measured delirium in-person (face-to-face) within the study according to standard diagnostic criteria no older than those in the Diagnostic and Statistical Manual of Mental Disorders, third edition (DSM-III) [23], the International Classification of Diseases, 10th revision (ICD-10) [24], or by validated delirium detection tools. Studies focusing on subsyndromal delirium, specific delirium subtypes (e.g., exclusively hyperactive delirium), or delirium due to intoxication or alcohol withdrawal were excluded. Studies were also excluded in which delirium detection relied solely on self- or proxy reports or on medical chart reviews of residents.

#### Context

Studies performed in nursing home settings were included. Nursing homes are defined as facilities that provide 24-h functional support for people who require assistance with activities/instrumental activities of daily living (ADL/IADL) and have identified health needs [25]. These facilities offer long-term care and may also provide post-acute, rehabilitation, palliative, and/or hospice care. Studies conducted in facilities that primarily provide care other than long-term residential care (e.g., skilled nursing facilities) were excluded.

#### Population

Studies conducted among residents of nursing homes were included. Studies that examined delirium exclusively in specific resident groups (e.g., residents with COVID-19 infection, acute illness, or following acute hospitalization) were excluded because they represent selective subsets of the nursing home population. Although dementia is highly prevalent in nursing homes, studies that included only residents with dementia were also excluded because a substantial proportion of nursing home residents do not have dementia [26]. Additionally, studies were excluded if they omitted groups typically appropriate for delirium assessment, such as residents without communication barriers.

#### Types of studies

Studies with observational designs, including cross-sectional and longitudinal studies, were eligible, including those providing cross-sectional prevalence data on delirium (e.g., baseline data from longitudinal studies). Studies were excluded if they reported prevalence data that did not represent the eligible study population. Additionally, studies that identified prevalence data through retrospective analysis (e.g., using preexisting research or routinely collected data) were excluded. To avoid potential bias from selective samples, studies with experimental designs (e.g., randomized controlled trials), case–control studies, or case series were excluded.

Furthermore, reviews, brief reports, conference abstracts, commentaries, letters to the editor, and publications in languages other than English or German were excluded.

### Study selection

Based on the results of the initial systematic searches, two reviewers (AHO and either RL or VM) independently conducted each screening step to identify eligible study reports. Any discrepancies were resolved via discussion. If consensus could not be reached, a third reviewer (JD) was consulted. To ensure consistent application of the eligibility criteria, an initial review of 100 titles and abstracts was conducted. Once consensus was achieved, all titles and abstracts were screened for eligibility. During the full-text screening, ten articles were initially screened to maintain consistency between the reviewers before assessing the remaining ones. For the updated search, study selection was independently conducted by AHO and RW.

### Outcome measures

The primary measure was the point prevalence of delirium, defined as the proportion (%) of residents identified as having delirium (*n* cases) out of the total number of residents assessed in each study (*n* total), based on cross-sectional data. The secondary measure was factors associated with the prevalence of delirium, if reported by the included studies. These factors were defined as significant variables (*p*-value ≤ 0.05) identified through multivariable analysis.

### Data extraction

The following data were extracted from the included studies: (1) General study characteristics included the first author’s last name, publication year, study aim, and design; (2) information about the eligible study population included the data collection year, country, study site(s), inclusion and exclusion criteria, sampling method, sample size, and participant characteristics; (3) information about the identification of delirium included the methods of measuring delirium, the individuals performing the measurements (rater), and the survey period (reference point); (4) the overall delirium prevalence data and stratified results (e.g., by sex, dementia); and (5) for studies reporting on associated factors, data on all comeasurements (e.g., variables, adjusted odds ratios [AOR]) at each level of analysis. Data extraction was independently performed by two reviewers (AHO and either RL or RW) using a standardized form, and any discrepancies were resolved via discussion. Missing data were calculated using appropriate formulas, such as by deriving proportions from available numerators and denominators.

### Quality assessment

The quality of the included studies was evaluated using the Joanna Briggs Institute (JBI) Critical Appraisal Checklist for Prevalence Data [22], which includes nine items that assess factors such as sample frame appropriateness and measurement validity (Supplementary Material 2). For this review, each item was rated as “Yes” (one point), “No” (zero points), or “Unclear” (zero points), resulting in a total score ranging from 0 to 9, with higher scores indicating lower risk of bias. For studies evaluating factors associated with the prevalence of delirium via multivariable analysis, the JBI Critical Appraisal Checklist for Analytical Cross-Sectional Studies [27] was used. This checklist includes eight items and assesses factors such as the presence of clearly defined inclusion criteria and the identification of confounding factors (Supplementary Material 2). The rating system for the items is identical to that used for the quality assessment of all studies. Item 4 (objective and standard criteria for measurement of the condition) was marked as “Not/Applicable” as diagnosis-based study populations were not eligible for this review. As a result, the total score in this review ranged from 0 to 7, with higher scores indicating lower risk of bias. The quality of the studies was evaluated independently by two reviewers (AHO and either RL or RW), and any discrepancies were resolved through discussion.

### Data synthesis

Data from individual studies were tabulated and key findings were summarized in the main text. A meta-analysis was conducted to pool the overall delirium prevalence data. Given the substantial heterogeneity among the included studies, a random-effects model was applied to appropriately synthesize the available prevalence data while acknowledging between-study variability [28]. Heterogeneity was assessed using the Wald-Chi2-Test (Wald-χ2) and I2 statistics. A leave-one-out sensitivity analysis was conducted to evaluate the influence of individual studies on the pooled estimate and heterogeneity. Publication bias was examined by assessing funnel plot asymmetry with Egger’s regression test [29]. Factors associated with the prevalence of delirium were grouped when definitions overlapped across studies, and these factors were then abstracted to a higher common level when appropriate. This approach also included factors that were not significantly associated with delirium in one study but significantly associated in another study, thus ensuring that all observations were captured.

## Results

### Study selection

**Fig. 1:**
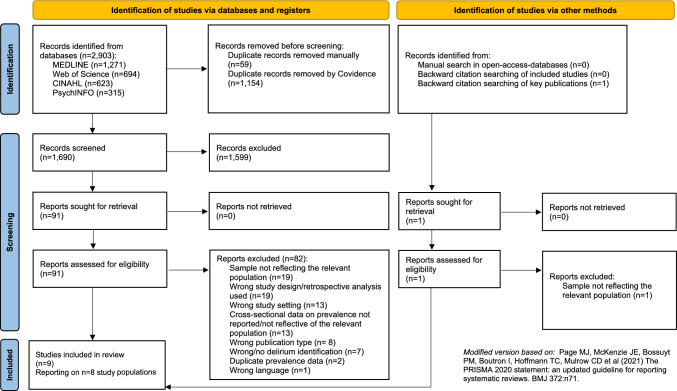
Flow diagram of the study selection process

The initial search of the electronic databases yielded 2648 records, and the updated search identified an additional 255 records. After excluding 1213 duplicates, 1690 records remained for title and abstract screening. Of these, 91 full-text articles were assessed for eligibility. Ten study reports were included for data extraction. However, two of these studies reported duplicate prevalence rates [30, 31]. Therefore, after data extraction, only the study most relevant to the objectives of this review was considered [31]. As a result, nine study reports were included in the final review [31–39]. Two of these reports [32, 35] were based on the same study sample but used different diagnostic criteria for delirium. Therefore, both studies were included in this review. However, to avoid bias from duplicate data, only the study with the most consistent and comprehensive reporting [32] was used for synthesizing sample-related characteristics (e.g., country, year of data collection, proportions). Consequently, eight studies with independent study populations were included in the synthesis of prevalence data [31–34, 36–39]. Four of these studies examined factors associated with the prevalence of delirium [31, 33, 36, 39], and were therefore included in the corresponding analysis. No additional studies were identified via manual searches. Figure 1 outlines the study selection and the reasons for exclusion.

### General characteristics

The included study reports (*n* = 9) [31–39] were published between 1998 and 2024. Five studies focused on the prevalence of delirium [31, 33, 36, 37, 39]. The remaining four studies addressed additional aspects of delirium research, including sensory impairments as associated factors for delirium across various settings [34], the validation of the 25-Item Delirium Observation Screening Scale (DOSS-25) [38], the comparison of delirium diagnostic criteria [32], and the long-term prognosis of delirium [35]. Six studies employed a cross-sectional approach [31, 32, 34, 36, 37, 39], while three used a longitudinal design [33, 35, 38], which included cross-sectional analyses. The general characteristics of all study reports are presented in Table 1.

**Table 1:** General and sample-related characteristics (studies sorted by sample size)

Source (year)	Primary focus	Study design	Sample-related characteristics								
			Country	Year of data	Sampling	Eligible criteria	Study sites (*n*)	Sample size (*n*)	Age, years	Female sex (%)	Dementia (%)
Morichi et al. [31] (2018)	Prevalence	C	Italy	2015	Cons.	• Inclusion: aged ≥ 65 years, native Italian speakers, written informed consent • Exclusion: coma, aphasia, end-of-life status as defined by clinical judgment	NHs (71)	1454	84.4 (mean)	69.8ª	51.9ᵇ
Fedecostante et al. [39] (2024)	Prevalence	C	Italy	2016	Cons.	• Inclusion: aged > 65 years • Exclusion: aphasia, blindness, deafness, critical conditions (coma, terminally ill)	NHs (32)	955	84.7 (mean)	69.8	43.8^c^
Morandi et al. [34] (2020)	Predictor analysis	C	Italy	2017	Cons.	• Inclusion: aged ≥ 65 years, provided informed consent • Exclusion: refusal to participate, severe visual impairment and deafness, aphasia, coma	NHs (N/R)	671^d^	N/R	N/R	N/R
Sabbe et al. [36] (2021)	Prevalence	C	Belgium	2018	Cons.	• Inclusion: aged ≥ 65 years, native Dutch speakers, written informed consent • Exclusion: coma, aphasia, end-of-life status (residents that were assumed to live only a couple of days), residents on specific dementia wards	NHs (6)	338	84.7 (mean)	67.5	27.5
McCusker et al. [33] (2011)	Incidence/Prevalence	L	Canada	Baseline: 2007^e^	Cons. (random sampling when needed^f^)	• Inclusion: aged ≥ 65 years, admitted for long-term care • Exclusion: unable to communicate in English or French, primary nurse or research assistant did not have time to complete assessments on additional residents because of a high workload	LTCF (7)	279	65–79: 22.9% 80–89: 49.8% 90+: 27.2%	56.3	65.5
Teale et al. [38] (2018)	Instrument validation	L	United Kingdom	Baseline: 2015	Cons.	• Exclusion: aged < 65 years, end-of-life or palliative care (as advised by care home staff), communication difficulties significant enough to preclude completion of CAM	NCHs (9)	216	84.9 (mean)	61	40
Sandberg et al. [37] (1998)	Prevalence	C	Mid-Sweden	N/R	Cons.	• Inclusion: aged ≥ 75 years, provided informed consent	NHs (3)	202	84.2 (mean)	65.3	66.3
Laurila et al. [32] (2003)^g^	Criteria match	C	Finland	1999–2000	Cons.	• Inclusion: provided informed consent • Exclusion: aged < 70 years, coma	NHs (7): 13 wards	195	> 85: 56.9%	90.8	86.2
Pitkala et al. [35] (2005)^g^	Prognostic factors	L	Finland	N/R	Cons.	• Inclusion: provided informed consent • Exclusion: aged < 70 years, coma	NHs (7): 13 wards	195	N/R	N/R	86

*C* cross-sectional study, *Cons.* consecutive sampling, *CAM* Confusion Assessment Method, *L* longitudinal study, *LTCF* long-term care facilities, *N/R* not reported, *NCHs* nursing care homes, *NHs* nursing homes

ªThe relative frequency of females stated (70.2% in a total sample of 1.454 residents) does not match the reported data in the stratified groups (*n* = 362 females with and *n* = 653 females without delirium). The calculation was therefore based on the stratified data: (362 + 653)/1.454 = 0.698

ᵇCalculation using information from the publication: 754/1.454 = 0.519

^c^Calculation using information from the publication: (232 + 186)/955 = 0.438

^d^The sample size (*n* = 667) does not match the reported relative frequency of delirium (32.3%) or the stratified data (*n* = 217 with and *n* = 454 without delirium in nursing homes). The sample size was therefore determined on the basis of the stratified data: 217 + 454 = 671

^e^Determination on the basis of information from the publication: February 2008 (last follow-up)—6 months follow-up (total) = August 2007

^f^“to achieve […] targeted 3:1 ratio of participants in Cohorts […] and to take into account the available time of research and nursing staff” [33]

^g^Both studies [32, 35] report results based on the same sample and data collection process

### Sample-related characteristics

Three study samples were drawn from Italy [31, 34, 39], whereas the remaining populations originated from Finland [32], Belgium [36], Canada [33], mid-Sweden [37] and the United Kingdom [38]. The oldest data reported were collected in 1999, and the most recent data were collected in 2018 (*n* = 7 studies) [31–34, 36, 38–40]. The sampling procedure followed a consecutive approach in all included studies. For the recruitment of one study [33], a random sampling approach was additionally applied. The number of facilities reported ranged from 3 to 71 (*n* = 7 studies) [31–33, 37–40], and the number of residents ranged from 195 to 1454 (*n* = 7 studies) [31–34, 37–40], comprising a total of 4291. All included residents were aged 65 years or above. The reported mean age of the residents ranged from 84.2 to 84.9 years (*n* = 5 studies) [31, 37–40]. In one study [33], 49.8% of the residents were aged 80 to 89 years, whereas in another study [32], 56.9% of the residents were over 85 years old. The reported proportion of female residents varied widely, ranging from 56.3% to 90.8% (*n* = 7 studies) [31–33, 37–40]. The percentage of residents with dementia was more heterogeneous, ranging from 27.5% to 86.2% (*n* = 7 studies) [31–33, 37–40]. A summary of all study populations is provided in Table 1.

### Characteristics of delirium detection

Delirium was primarily assessed via the 4 A’s test (4AT) (*n* = 3 studies) [31, 34, 39] and the 4-item Confusion Assessment Method (CAM-4) (*n* = 2 studies) [33, 38]. Furthermore, the 13-item DOSS (DOSS-13) [36] and Organic Brain Syndrome Scale (OBS) [37] were each used in one study, whereas the DSM-IV [35] and DSM-III-R [32] criteria were applied in a common study population. According to the studies, delirium was assessed by physicians or nurses directly involved in resident care (*n* = 4 studies) [31, 32, 34, 39] as well as by research personnel (*n* = 4 studies) [33, 36–38]. For the detection of delirium in four studies [32, 36–38], the raters who conducted the assessments were reported to be trained. The period in which the cross-sectional data were collected was reported to range from one day to 11 months (*n* = 7 studies) [31–34, 37, 39, 40]. All information regarding the delirium detection is provided in Table 2.

**Table 2:** Delirium detection and prevalence (studies sorted by sample size)

Source (year)	Sample size (*n*)	Delirium (%)	Delirium detection			Delirium prevalence by subgroups			Dementia (%)	Risk of bias scoreª
			Delirium measure	Person measuring (rater)	Point reference	Age, years (%)	Sex (%)	Dementia status (%)		
Morichi et al. [31] (2018)	1454	36.8	4AT	Attending physicians, mainly geriatricians with long-term work experience in NHs	1 day (30.09.2015)	N/R	F: 35.7^b^ NF: 39.4^d^	Y: 53.2 N: 19.1	51.9^c^	7/9
Fedecostante et al. [39] (2024)	955	27.2	4AT	Physicians or nurses who were involved in daily care of enrolled residents	1 day (28.09.2016)	N/R	F: 28.8^e^ NF: 23.6^ h^	Y: 44.5^f^ N: 13.8^i^	43.8^g^	7/9
Morandi et al. [34] (2020)	671^j^	32.3	4AT	Attending physicians	1 day (in 2017)	N/R	N/R	N/R	N/R	6/9
Sabbe et al. [36] (2021)	338	14.2	DOSS-13	Trained research nurses (*n* = 3)	2 months (February–March 2018)	N/R	F: 14.0^k^ NF: 14.5^m^	Y:23.7^l^ N: 10.6^n^	27.5	8/9
McCusker et al. [33] (2011)	279	11.5	CAM-4	Research assistants, supervised by study psychiatrist	1 week (in August 2007^o^)	65–79: 6.3 80–89: 14.4 90+: 10.5	F: 11.5 NF: 11.5	Y: 16.5 N: 2.1	65.5	7/9
Sandberg et al. [37] (1998)	202	57.9	OBS with DSM-III-R consensus	Trained research assistants (*n* = 7) with experience of geriatric psychiatry, results consented by coauthors (*n* = 3)	3 months	N/R	N/R	Y: 63.5^p^ N: 46.9^q^	66.3	7/9
Teale et al. [38] (2018)	197	1.0^v^	CAM-4	Trained research assistants	N/R	N/R	N/R	N/R	40	7/9
Laurila et al. [32] (2003)^w^	195	14.4	DSM-III-R	Trained nurses most familiar to each patient, experienced in the care of patients with cognitive impairment and its testing, checked by experienced geriatricians (*n* = 2)	11 months (November 1999–October 2000)	N/R	N/R	N/R	86.2	7/9
Pitkala et al. [35] (2005)^w^	195	15.9	DSM-IV	N/R	N/R	N/R	N/R	N/R	86	5/9

*4AT* 4 A’s test, *CAM-4* 4-item Confusion Assessment Method, *DOSS-13* 13 Item Delirium Observation Screening Scale, *DSM-III-R* Diagnostic and Statistical Manual of Mental Disorders, Third Edition, Revision, *DSM-IV* Diagnostic and Statistical Manual of Mental Disorders, Fourth Edition, *F* Female, *JBI* Joanna Briggs Institute, *N* no, *N/R* not reported, *NF* nonfemale, *NHs* nursing homes, *OBS* Organic Brain Syndrome Scale, *Y* yes

ªThe score is based on a scale of 1 to 9 points (higher scores indicate lower risk of bias), determined using the JBI Critical Appraisal Checklist for Studies Reporting Prevalence Data [22] (Supplementary Material 2)

^b^Calculation using information from the publication: 362/(362 + 653) = 0.357

^c^Calculation using information from the publication: 754/1.454 = 0.519

^d^Calculation using information from the publication: (535–362)/((535–362) + (919–653)) = 0.394

^e^Calculation using information from the publication: 192/667 = 0.288

^f^Calculation using information from the publication: 186/(232 + 186) = 0.445

^g^Calculation using information from the publication: (232 + 186)/955 = 0.438

^h^Calculation using information from the publication: (260–192)/(955–667) = 0.236

^I^Calculation using information from the publication: (260–186)/((260–186) + (695–232)) = 0.138

^j^The sample size (*n* = 667) does not match the reported relative frequency of delirium (32.3%) or the stratified data (*n* = 217 with and *n* = 454 without delirium in nursing homes). The sample size was therefore determined on the basis of the stratified data: 217 + 454 = 671

^k^Calculation using information from the publication: 32/228 = 0.140

^l^Calculation using information from the publication: 22/93 = 0.237

^m^Calculation using information from the publication: 16/110 = 0.145

^n^Calculation using information from the publication: (48–22)/(338–93) = 0.106

^o^Determination using information from the publication: February 2008 (last follow-up)—6 months follow-up (total) = August 2007

^p^Calculation using information from the publication: ((42.1 * 202)/100)/((66.3 * 202)/100) = 0.635

^q^ Calculation using information from the publication: (((57.9 * 202)/100)  −  ((42.1 * 202)/100))/(202 − ((66.3 * 202)/100)) = 0.469

^v^CAM was only used for 197 (of 216) participants at baseline, of which 2 were positive: 2/197 = 0.010

^w^Both studies [32, 35] report results based on the same sample and data collection process

### Quality assessment of the included studies

The overall quality assessment of all studies (*n* = 9) [31–39] is presented in Table 2**,** with item-level details provided in Supplementary Material 3. The quality ratings for the eight studies included in the synthesis of the prevalence data on delirium [31–34, 36–39] ranged from 6 to 8 out of 9 possible points. The main risk of bias identified was incomplete statistical reporting in 8/8 studies [31–34, 36–39], which in all cases was due to the lack of reporting on confidence intervals for the prevalence estimates. Additionally, the quality of the four studies reporting factors associated with the prevalence of delirium [31, 33, 36, 39] was assessed; the overall findings are presented in Table 3, with further item-level details provided in Supplementary Material 4. The quality ratings ranged from 4 to 6 out of a possible 7 points. The main risk of bias reported was unclear reliability of delirium measurement (3/4 studies) [31, 33, 39].

**Table 3:** Categorization of associated factors across studies (including non-associated factors in the case of overlapping definitions)

Source (year)	Delirium measure (prevalence)	Measurement or source	Variable	Estimation (95%-CI)	*p* value	Associated factor	Categorization (subcategory)	Risk of bias scoreª
Morichi et al. [31] (2018)	4AT (36.8%)	KI^b^	ADL: 0 functions spared	AOR^c^ 6.13 (3.08–12.19)	< 0.001	Yes	ADL impairment (severe)	6/7
			ADL: 1–5 functions spared (ref. ADL: 6 functions spared)	AOR^c^ 1.99 (1.03–3.84)	0.040	Yes	ADL impairment (moderate)	
		Medical record and/or prescribed acetylcholinesterase inhibitors or memantine recorded	Dementia	AOR^c^ 3.12 (2.38–4.09)	< 0.001	Yes	Cognitive impairment (Dementia)	
		Use recorded	Physical restraints	AOR^c^ 2.48 (1.71–3.59)	< 0.001	Yes	Physical restraints	
		Medical record	Antipsychotics	AOR^c^ 2.40 (1.81–3.18)	< 0.001	Yes	Antipsychotics	
		Clinical judgment of attending physician	Nutritional status: malnourished	AOR^c^ 4.87 (2.68–8.84)	< 0.001	Yes	Nutritional status (malnourished)	
			Nutritional status: at risk of malnutrition (ref. Nutritional status: well nourished)	AOR^c^ 1.16 (0.87–1.55)	0.317	No	Nutritional status (risk of malnutrition)	
		Years of study	Education	AOR 0.94 (0.91–0.97)	0.001	Yes	Education	
Fedecostante et al. [39] (2024)	4AT (27.2%)	KI^b^	ADL: 0 functions spared	AOR^d^ 4.14 (1.14–15.11)	0.032	Yes	ADL impairment (severe)	5/7
			ADL: 1–5 functions spared (ref. ADL: 6 functions spared)	AOR^d^ 1.26 (0.36–4.39)	0.722	No	ADL impairment (moderate)	
		Medical record and/or prescribed acetylcholinesterase inhibitors or memantine recorded	Dementia	AOR^d^ 3.34 (2.34–4.77)	< 0.001	Yes	Cognitive impairment (Dementia)	
		Use recorded	Physical restraints	AOR^d^ 1.77 (1.23–2.57)	0.002	Yes	Physical restraints	
		Medical record	Antipsychotics	AOR^d^ 2.03 (1.43–2.87)	< 0.001	Yes	Antipsychotics	
		Medical record	Antidepressants SSRIs	AOR^d^ 0.64 (0.42–0.99)	0.043	Yes	N/A^e^	
		Clinical judgment of attending physician	Nutritional status: malnourished	AOR^d^ 4.22 (1.78–9.99)	0.001	Yes	Nutritional status (malnourished)	
			Nutritional status: at risk of malnutrition (ref. Nutritional status: well nourished)	AOR^d^ 2.28 (1.57–3.30)	< 0.001	Yes	Nutritional status (risk of malnutrition)	
		Years of study	Education	AOR^d^ 0.90 (0.85–0.95)	< 0.001	Yes	Education	
Sabbe et al. [36] (2021)	DOSS-13 (14.2%)	KI^b^	ADL: score (4–24)	OR^f^ 1.12 (1.05–1.20)	N/R	No	ADL impairment	5/7
		MoCA^g^	Cognitive impairments: score (0–30)	AOR 0.69 (0.63–0.77)	N/R	Yes	Cognitive impairment	
		Medical record	Dementia	OR^f^ 2.61 (1.39–4.89)	N/R	No	Cognitive impairment (Dementia)	
		Visually determined	Being physically restrained	OR^f^ 3.52 (1.76–7.06)	N/R	No	Physical restraints	
		Medical record	Antipsychotics	OR^f^ 2.65 (1.31–5.38)	N/R	No	Antipsychotics	
		Medical record	Fall incident < 90 days	AOR 2.76 (1.24–6.14)	N/R	Yes	N/A^e^	
McCusker et al. [33] (2011)	CAM-4 (11.5%)	BI^h^	ADL impairment: severe (0–19)	AOR^i^ 8.39 (1.42–49.75)	0.01 ≤ *p* < 0.05	Yes	ADL impairment (severe)	4/7
			ADL impairment: moderate (20–59) (ref. ADL impairment: mild to none [≥ 60])	AOR^i^ 1.36 (0.24–7.71)	N/R	No	ADL impairment (moderate)	
		HDS^j^	Severe (0–39)	AOR^i^ 7.56 (1.68–33.91)	< 0.01	Yes	Cognitive impairment	
			Moderate (40–99) (ref. mild to none [≥ 100])	AOR^i^ 7.70 (1.99–29.84)	< 0.01	Yes	Cognitive impairment	
		Medical record	Dementia	AOR^i^ 5.84 (1.12–30.53)	0.01 ≤ *p* < 0.05	Yes	Cognitive impairment (Dementia)	
		Medical record	Hypertension	AOR^i^ 4.26 (1.27–14.32)	0.01 ≤ *p* < 0.05	Yes	N/A^e^	
		CSDD^k^	Depressive symptoms	AOR^i^ 3.43 (1.09–10.80)	0.01 ≤ *p* < 0.05	Yes	N/A^e^	

*4AT* 4 A’s test, *ADL* activities of daily living, *AOR* adjusted odds ratio, *BI* Barthel Index for activities of daily living, *CAM-4* 4-item Confusion Assessment Method, *CI* confidence interval, *CSDD* Cornell Scale for depression in dementia, *DOSS-13* 13-Item Delirium Observation Screening Scale, *HDS* Hierarchic Dementia Scale, *JBI* Joanna Briggs Institute, *KI* Katz Index of independence in activities of daily living, *MoCA* Montreal Cognitive Assessment, *N/A* not applicable, *N/R* not reported, *OR* odds ratio, *ref.* reference

ªThe score is based on a scale of 1 to 7 points (higher scores indicate lower risk of bias), determined using the JBI Critical Appraisal Checklist for Analytical Cross-Sectional Studies [27] (Supplementary Material 2)

^b^KI measures ADL on a 0–6 scale (higher scores indicate greater dependency) [76]. Sabbe et al. used a 6–24 scale [36]

^c^Further nonsignificant or noncomparable adjustments: Male sex, age (years), number of drugs, diuretics, antihypertensive drugs, statins/hypolipidemic drugs, antiulcer drugs, antidepressants, and urinary catheters

^d^Further nonsignificant or noncomparable adjustments: Age (years), sex, atypical antidepressants (including trazodone), polypharmacy (≥ 5 drugs). Variables for the model were selected via the “stepwise backward” method

^e^Grouping was not possible because only a single measurement was used in one study

^f^Variable included in univariate regression but not in the multivariate model. Variables for the model were selected via the “stepwise forward” method

^g^MoCA measures cognitive impairment on a scale ranging from 0–30 (lower scores indicate greater impairment) [77]

^h^BI measures ADL on a scale ranging from 0–100 (lower scores indicate greater dependency) [78]

^i^Further nonsignificant or noncomparable adjustments: Age (65–79 as ref.; 80–89; 90+), sex (female as ref.; male), and time since admission (< 1 year; 1 year or more as ref.)

^j^HDS measures cognitive impairment on a scale ranging from 0–200 (lower scores indicate greater impairment) [79]

^k^CSDD measures depressive symptoms in dementia [80]; ≥ 6 points indicate depressive symptoms[81]

### Prevalence of delirium in nursing home residents

The overall prevalence of delirium in the included studies (*n* = 8) [31–34, 36–39] varied widely from 1.0% to 57.9%. All results concerning the prevalence of delirium are summarized in Table 2. For the two most commonly used delirium detection tools, delirium prevalence rates ranged from 27.2% to 36.8% for the 4AT (*n* = 3 studies) [31, 34, 39] and from 1.0% to 11.5% for the CAM-4 (*n* = 2 studies) [33, 38]. The latter method yielded the lowest prevalence of delirium in the entire data corpus. In contrast, the greatest prevalence of delirium (57.9%) was observed via the OBS [37]. Among residents with dementia, the prevalence of delirium ranged from 16.5% to 63.5%; among residents without dementia, the prevalence of delirium ranged from 2.1% to 46.9% (*n* = 5 studies) [31, 33, 36, 37, 39]. In each of these five studies, the prevalence of delirium was consistently greater in residents with dementia than in those without dementia.

### Meta-analysis of the overall prevalence rates

**Fig. 2:**
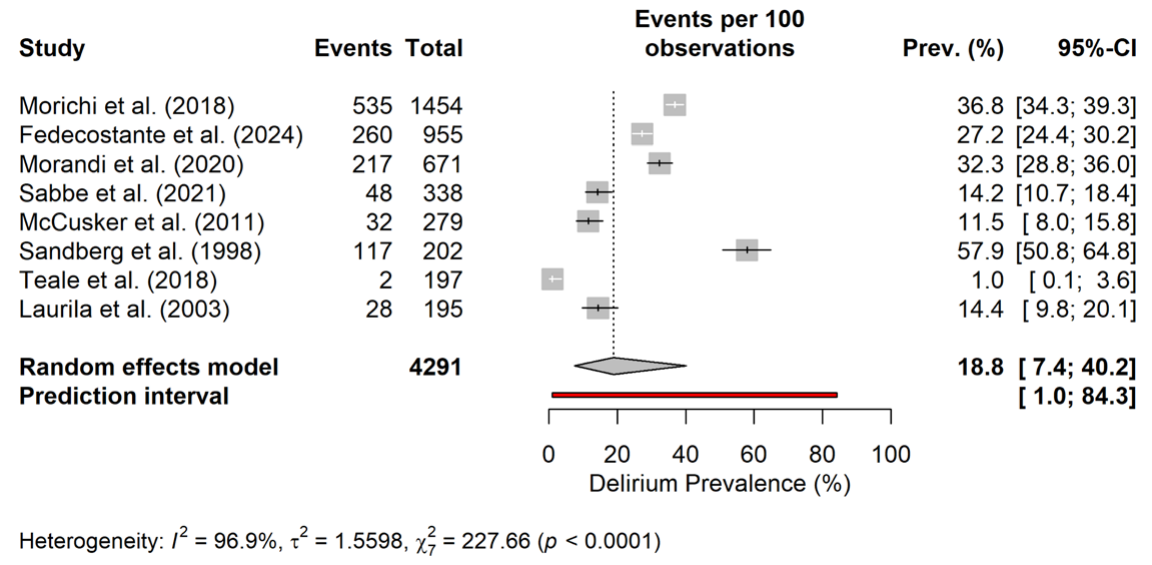
Forest plot from the meta-analysis of overall delirium prevalence

**Fig. 3:**
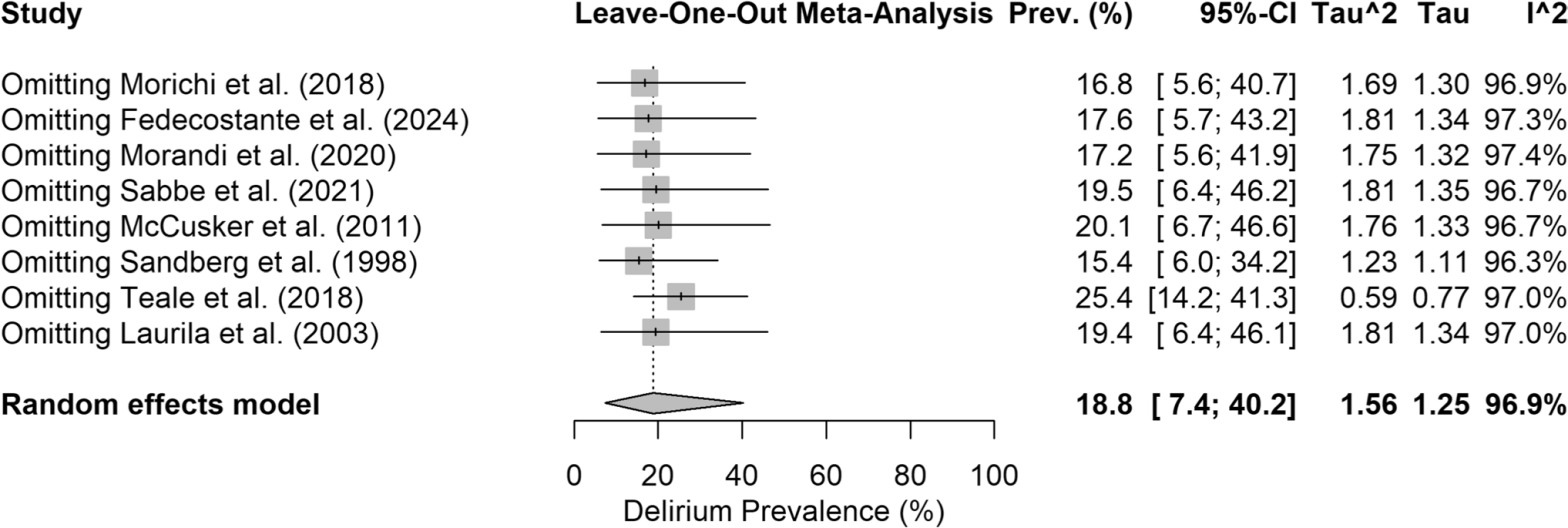
Forest plot of the leave-one-out sensitivity analysis of the overall results

**Fig. 4:**
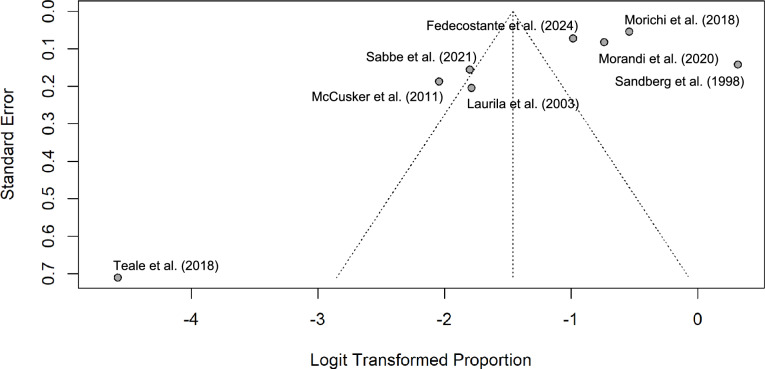
Funnel plot showing the assessment of potential publication bias

The pooled prevalence of delirium across the included studies (*n* = 8) [31–34, 36–39], encompassing 4291 residents, was 18.8% (95% confidence interval (CI): 7.4%–40.2%). Considerable heterogeneity existed between the studies (I2 = 96.9%; Wald-χ^2^_7_ = 227.66, *p* < 0.001). Figure 2 presents a forest plot illustrating the findings. The exclusion of individual study reports did not lead to notable changes in heterogeneity or pooled estimates, as illustrated in Fig. 3. To assess a potential publication bias, a funnel plot was evaluated as shown in Fig. 4. Egger’s test indicated no significant evidence of publication bias (*t* = −1.82; *p* = 0.119).

### Associations with the prevalence of delirium

Multivariable regression analyses were conducted in four studies [31, 33, 36, 39] to identify factors associated with the prevalence of delirium. A total of 23 factors were found to be associated and were categorized when appropriate, along with seven non-associated factors. The detailed results of the included factors, including their measurements, variables, and estimates, are presented in Table 3. Cognitive impairment was found to be associated with the prevalence of delirium in nursing home residents in all four studies that examined this association [31, 33, 36, 39]. In particular, dementia was identified in three [31, 33, 39] of the four studies as an associated factor. ADL impairment was also found to be associated in three [31, 33, 39] of the four studies. Severe ADL impairment was associated in all three of these studies, while moderate impairment showed an association in one [31] of these three studies. Nutritional status was associated in both studies in which it was assessed [31, 39]. Malnutrition showed an association in both studies, whereas the risk of malnutrition was associated in one [39] of those two. Furthermore, the use of physical restraints and administration of antipsychotics were each found to be associated in two [31, 39] of three studies [31, 36, 39] examining these factors. Education was identified as a protective factor in both studies that examined this variable [31, 39]. Additional factors that were found to be associated with the delirium prevalence in individual studies were falls within the last 90 days [36], depressive symptoms, hypertension [33], and the administration of antidepressants (SSRIs) as a protective factor [39].

## Discussion

The primary aim of this review was to assess the prevalence of delirium in the nursing home population. The results revealed that the prevalence of delirium ranged from 1.0% to 57.9%, with a pooled estimate of 18.8% (95% CI: 7.4%−40.2%). As all included studies examined residents aged 65 years and above, the findings reflect the prevalence of delirium among older nursing home residents. Overall, delirium emerged as a common health concern in this population, with a pooled prevalence of delirium that is slightly lower than the pooled estimate of 23.6% observed in hospitalized older adults [41]. In nursing homes, nurses play a pivotal role in early recognition owing to their continuous presence, whereas physician availability is less consistent in this setting than in acute care. Nevertheless, delirium is often under-recognized by nursing staff in nursing homes, particularly due to the symptom overlap with comorbid dementia [42]. This overlap is particularly relevant because the prevalence of delirium superimposed on dementia in nursing home residents reached a maximum of 63.5% in the included studies. Unrecognized delirium is associated with adverse consequences, including greater mortality [43, 44]. Therefore, strengthening the recognition skills of nursing staff via targeted education and the standardized use of delirium detection tools is critical to improving care.

The findings of this review differ from those of a recent narrative review, which reported that the prevalence of delirium ranged from 1.4%−70.3% [19]. This broad range in prevalence was likely driven by the inclusion of studies with heterogeneous methods and resident subgroups. In contrast, our review focused on minimally selected study populations, applied consistent methodological criteria, and provided a pooled point prevalence estimate (18.8%). Nevertheless, prevalence estimates still varied substantially (1.0%−57.9%), and heterogeneity was very high across the studies (e.g., I2 = 96.9%). This variability limits the precision of the pooled estimate and may have been due to additional factors, such as further differences in study populations and detection approaches. Sensitivity analyses excluding individual outlier studies [37, 38] did not materially reduce this inconsistency. With only eight studies included in the meta-analysis, further investigation of heterogeneity via subgroup analyses or meta-regression was not meaningful. Therefore, potential sources of variation are narratively discussed.

### Variations among the study populations

Considerable variability was observed across the included study populations, particularly in the prevalence of dementia, which ranged from 27.5% [36] to 86.2% [32]. These differences likely contributed to the variability in delirium rates because dementia is a well-established major risk factor for delirium [2]. The findings of this review support this contribution: the prevalence of delirium is consistently greater among residents with dementia than those without dementia [31, 33, 36, 37]. In addition, further differences across the samples can be identified, although these differences were not systematically examined within the scope of this review. For example, antipsychotic administration, the use of physical restraints [36, 39], and the presence of visual or hearing impairments among residents [33, 37] differed across samples, and these factors have all been reported in the literature as risk factors for delirium [2]. One important factor contributing to these differences may be variations in how nursing homes are defined across countries. For example, nursing homes may differ in the types of care they provide, such as whether subacute care is offered in addition to long-term care, as well as in the level of assistance provided, physician coverage, and the availability of trained staff [25, 45]. However, these differences cannot be further assessed based on the available data.

### Conceptual variations in detection methods

Differences in how delirium is defined may also have contributed to the observed heterogeneity. In the included studies, the 4AT, CAM-4, DOSS-13, OBS, and DSM-III-R criteria were used to detect delirium, with research conducted over a broad period from 1998 to 2024 [31–34, 36–39]. The understanding of delirium has changed over the more than 30 years since its initial definition in the DSM-III criteria to the current DSM-5-TR [1, 23, 46–49]. These changes are reflected in the inclusiveness of the diagnostic criteria: In a sample of hospital patients and residents of nursing homes, Laurila et al. [32] found that the DSM-IV criteria were the most inclusive, identifying 24.9% of participants with delirium, followed by DSM-III-R (19.5%) and DSM-III (18.8%). Adamis et al. [50] reported a prevalence rate of 19.5% using DSM-IV criteria in older hospital patients, compared to 13.0% using DSM-5 criteria. These differences are relevant because the development of most of the detection tools used was influenced by the respective DSM criteria. For example, the CAM [51] is based on DSM-III-R criteria, the DOSS [52] is based on DSM-IV criteria, and the 4AT [53] was developed when DSM-IV-TR was still in use. However, among these instruments, the CAM appears to be the most robust to changes because it is the only detection tool that reflects the more recent DSM-5 criteria [54].

Current research suggests that delirium can best be characterized by three symptom domains: cognitive (e.g., orientation and attention), higher-order thinking (e.g., language and thought process), and circadian (e.g., sleep–wake cycle disturbances) [3–7]. Using these domains as a reference reveals further content-related differences: Although the 9-item version of the CAM covers all three domains, the CAM-4, as used in this review, omits the circadian domain, and the 4AT omits higher-order thinking [55]. This omission is also important as symptoms reflecting these domains may be relevant indicators for distinguishing delirium from dementia [56].

### Delirium detection in dementia populations

The 4AT, CAM, and DOSS have generally demonstrated good diagnostic performance across various clinical populations [50–52]. However, studies suggest that the accuracy of both the CAM and the 4AT may be reduced in populations with a higher prevalence of dementia [7, 53, 57–59]. For the 4AT, this reduction may particularly be due to items 2 and 3 (Abbreviated Mental Test-4 [60] and Months Backwards test [61]), which assess cognitive functions frequently impaired in dementia [62], and item 1 (alertness), which conflates arousal and motor behavior and may therefore complicate sensitive assessment in cognitively impaired individuals [7].

The study using the OBS reported the highest prevalence of delirium in this review at 57.9% and also showed a similarly high prevalence of dementia [37]. The OBS was originally developed for the evaluation of dementia [63] but has also been regarded as an instrument for delirium detection [63–65] and has demonstrated some evidence of concurrent validity with the CAM [66]. However, it has not been formally validated as a delirium-specific instrument, and its ability to distinguish delirium from dementia has not been systematically investigated.

DOSS-13 is the only tool in this review that has been validated for use in long-term care settings. Sabbe et al. [40] reported partially excellent test properties for the DOSS-13 in the same nursing home sample in which they assessed the delirium prevalence rate of 14.2% [36]. However, the dementia rate was only 27.5%, which limits the generalizability to nursing home populations with greater dementia rates. In contrast, other tools in the literature, such as the 4-DSD or the Ultra-Brief CAM (UB-CAM), have shown promising accuracy in samples with higher dementia prevalence [67]. However, as these tools have not been validated in nursing homes, their performance in settings with a high proportion of severe dementia remains uncertain. This highlights the need for robust validation studies of appropriate tools for nursing home settings.

### Administration of the detection methods

Across the included studies, variation can be observed in the qualification and training of rater groups. When using the 4AT, delirium screenings were most frequently performed by attending physicians [31, 34], but also by nursing staff directly involved in the care of residents [39]. In both CAM studies [33, 38], delirium assessments were performed by research assistants. However, in McCusker et al. [33], whether the raters had received CAM training was unclear, despite such training being recommended to ensure accuracy [68]. In addition, two studies employed rater groups with differing professions within the same study [37, 39]. This variability in the characteristics and training of raters could partly explain the heterogeneity in reported prevalence estimates of delirium. In this context, these findings underscore the importance of standardized procedures for delirium detection in nursing home settings. To ensure effective delirium detection, the qualifications and training level of professional groups should therefore be taken into account when introducing delirium detection tools into routine care practice [55].

### Associated factors with the delirium prevalence

Cognitive impairments, particularly dementia, and impairments in ADLs are reported in the literature as well-established risk factors for the development of delirium, regardless of the care setting [2]. In this review, both factors were proven to be particularly relevant because they were most frequently found to be associated with the prevalence of delirium [31, 33, 36, 39]. Many of the identified factors are described in the literature as interrelated, which may further increase the likelihood of delirium when they co-occur. For example, residents with dementia are more likely to be physically restrained [69], receive antipsychotic medications [70], or suffer from malnutrition [71]—all of which were associated with the prevalence of delirium in more than one of the included studies [31, 39]. Moreover, the administration of physical restraints and antipsychotics may themselves contribute to further cognitive and functional decline in residents [72, 73], potentially creating a self-reinforcing cycle of delirium risk. In contrast, factors such as falls [36], hypertension, or depressive symptoms [33] were each identified as relevant in only one included study and therefore appear less robust in this analysis. Overall, the findings of this review confirm the multifactorial nature of delirium and underscore the need for targeted, multidimensional prevention strategies, particularly for residents with dementia.

### Strengths and limitations

This work presents a systematic review and meta-analysis that provides evidence on the delirium prevalence among nursing home residents. Preregistered on PROSPERO and conducted according to PRISMA guidelines [21], it employed a comprehensive multidatabase search with independent review at all stages. By applying consistent methodological criteria, it provides the first pooled estimate describing how common delirium is within this population.

However, this review has several limitations. First, despite eligibility criteria designed to address this issue, the included studies exhibited considerable heterogeneity. In addition, the limited number of relevant studies further restricts the precision of the pooled prevalence estimate and precludes a more detailed quantitative assessment of heterogeneity from being meaningful [74]. Part of this heterogeneity may be attributable to the second limitation: the eligibility criteria did not distinguish between formal diagnostic criteria, diagnostic tools, or screening instruments, nor did they account for how these methods were applied. For example, whether delirium was diagnosed via the use of DSM criteria by a trained psychiatrist or identified using a low-threshold screening tool administered by bedside nurses may affect delirium detection rates [75]. Third, all included studies examined nursing home residents aged ≥ 65 years. Therefore, the findings cannot be extended to younger residents. Moreover, most samples were drawn from European countries, with only one study originating from Canada, which may further limit the generalizability of the findings. Fourth, the prevalence estimates based solely on cross-sectional data can lead to an underestimation of delirium, as the fluctuating course of delirium may not have been fully captured. Fifth, the use of cross-sectional data also prevents any conclusions about causality regarding the factors found to be associated with the prevalence of delirium. Therefore, longitudinal study designs would be more appropriate. Sixth, the identification of factors associated with the prevalence of delirium was limited to studies that provided prevalence data and were therefore included in the review. As a result, relevant studies that examined associated factors but did not report delirium prevalence rates may have been overlooked, potentially leading to a biased or incomplete representation of the evidence.

## Conclusion

This review revealed that the point prevalence of delirium among older nursing home residents (aged ≥ 65 years) ranged widely from 1.0% to 57.9%. The pooled estimate was 18.8%, although heterogeneity across the studies was considerable, most likely due to differences in the prevalence of dementia and delirium detection methods. Cognitive impairments, particularly dementia, were most frequently associated with delirium. Despite the heterogeneity in the reported prevalence rates, delirium emerged as a common health issue across most studies. Future research should focus on developing and evaluating strategies for the timely detection and prevention of delirium, specifically tailored to the nursing home setting. Given the central role of nursing staff in detecting acute changes in residents’ mental status, targeted staff education and training are essential for early delirium recognition. Efforts are needed to develop and validate detection tools for populations with a high prevalence of dementia, particularly in the nursing home setting, and grounded in a commonly agreed understanding of delirium. More methodologically robust and internationally comparable prevalence assessments are also required to enable more reliable estimates for this vulnerable population.

## Supplementary Information

Below is the link to the electronic supplementary material.

Supplementary file1 (DOCX 59 KB)

## Data Availability

Data available within the article or its supplementary materials.

## Abbreviations

4AT: 4 A’s test
ADL: Activities of daily living
AOR: Adjusted odds ratio
CAM: Confusion Assessment Method
CAM-4: 4-Item Confusion Assessment Method
CI: Confidence interval
DOSS: Delirium Observation Screening Scale
DOSS-13: 13-Item Delirium Observation Screening Scale
DSM-III: Diagnostic and Statistical Manual of Mental Disorders, Third Edition
DSM-III-R: Diagnostic and Statistical Manual of Mental Disorders, Third Edition, Revision
DSM-IV: Diagnostic and Statistical Manual of Mental Disorders, Fourth Edition
DSM-IV-TR: Diagnostic and Statistical Manual of Mental Disorders, Fourth Edition, Text Revision
DSM-5: Diagnostic and Statistical Manual of Mental Disorders, Fifth Edition
DSM-5-TR: Diagnostic and Statistical Manual of Mental Disorders, Fifth Edition, Text Revision
JBI: Joanna Briggs Institute
OBS: Organic Brain Syndrome Scale
PRISMA: Preferred Reporting Items for Systematic Reviews and Meta-Analyses
PROSPERO: International Prospective Register of Systematic Reviews
Wald-χ2: Wald-Chi2-Test
