# Supplementary material for: Prevalence of delirium among older nursing home residents: a systematic review and meta-analysis
Source: Eur Geriatr Med. 2026 Feb 19;17(3):1357–76. doi: 10.1007/s41999-026-01422-0 (PMC13309485; doi:10.1007/s41999-026-01422-0)
Supplement: Supplementary file 1 — Supplementary file1 (DOCX 59 KB) [file 41999_2026_1422_MOESM1_ESM.docx]

**Supplementary Material 1: Example of the search strategy for Medline (via PubMed)**

| Search number | Search strategy (no time limit) |
| --- | --- |
| Basic search for “delirium” | |
| # 1 | Delirium[MeSH] |
| # 2 | delir*[Title/Abstract] |
| # 3 | Confusion[MeSH] |
| # 4 | confus*[Title/Abstract] |
| # 5 | "acute brain fail*"[Title/Abstract] |
| # 6 | "acute brain dysfunction*"[Title/Abstract] |
| # 7 | "organic brain syndrome*"[Title/Abstract] |
| # 8 | "altered mental stat*"[Title/Abstract] |
| # 9 | **1 OR 2 OR 3 OR 4 OR 5 OR 6 OR 7 OR 8** |
| Basic search for “nursing home” | |
| # 10 | Residential facilities[MeSH] |
| # 11 | "residential facilit*"[Title/Abstract] |
| # 12 | "residential home*"[Title/Abstract] |
| # 13 | "nursing facilit*"[Title/Abstract] |
| # 14 | "nursing home*"[Title/Abstract] |
| # 15 | "care facilit*"[Title/Abstract] |
| # 16 | "care home*"[Title/Abstract] |
| # 17 | Long Term Care[MeSH Terms] |
| # 18 | "long term car*"[Title/Abstract] |
| # 19 | “residential car*”[Title/Abstract] |
| # 20 | "assisted living*"[Title/Abstract] |
| # 21 | **10 OR 11 OR 12 OR 13 OR 14 OR 15 OR 16 OR 17 OR 18 OR 19 OR 20** |
| Basic search for “prevalence” | |
| # 22 | Epidemiologic Methods[MeSH] |
| # 23 | epidemiolog*[Title/Abstract] |
| # 24 | morbid*[Title/Abstract] |
| # 25 | Occurrence[MeSH]] |
| # 26 | occur*[Title/Abstract] |
| # 27 | frequen*[Title/Abstract] |
| # 28 | inciden*[Title/Abstract] |
| # 29 | prevalen*[Title/Abstract] |
| # 30 | **22 OR 23 OR 24 OR 25 OR 26 OR 27 OR 28 OR 29** |
| # 31 | **9 AND 21 AND 30** |

**Supplementary Material 2: Items for the critical appraisal of the included studies**

| **Critical Appraisal Checklist for Studies Reporting Prevalence Data [21]^a^**  *1. Was the sample frame appropriate to address the target population?*  *2. Were study participants recruited in an appropriate way?*  *3. Was the sample size adequate?*  *4. Were the study subjects and setting described in detail?*  *5. Was data analysis conducted with sufficient coverage of the identified sample?*  *6. Were valid methods used for the identification of the condition?*  *7. Was the condition measured in a standard, reliable way for all participants?*  *8. Was there appropriate statistical analysis?*  *9. Was the response rate adequate, and if not, was the low response rate managed appropriately?*  Possible responses: “Yes”, “No”, “Unclear” and “Not applicable” |
| --- |
| **Critical Appraisal Checklist for Analytical Cross Sectional Studies [25]^b^**  *1. Were the criteria for inclusion in the sample clearly defined?*  *2. Were the study subjects and the setting described in detail?*  *3. Was the exposure measured in a valid and reliable way?*  *4. Were objective, standard criteria used for measurement of the condition?*  *5. Were confounding factors identified?*  *6. Were strategies to deal with confounding factors stated?*  *7. Were the outcomes measured in a valid and reliable way?*  *8. Was appropriate statistical analysis used?*  Possible responses: “Yes”, “No”, “Unclear” and “Not applicable” |

© JBI, 2020. All rights reserved. JBI grants use of these tools for research purposes only.

^a^ Munn Z, Moola S, Lisy K, Riitano D, Tufanaru C (2015) Methodological guidance for systematic reviews of observational epidemiological studies reporting prevalence and cumulative incidence data. JBI Evidence Implementation 13:147-153.

^b^ Moola S MZ, Tufanaru C, Aromataris E, Sears K, Sfetcu R, Currie M, Qureshi R, Mattis P, Lisy K, Mu P-F. (2020) Systematic reviews of etiology and risk. In: Aromataris E MZ (ed) JBI Manual for Evidence Synthesis JBI.

**Supplementary Material 3: Quality assessment of all included studies reporting prevalence data**

| Source (year) | Criteria and corresponding ratings^a^ | | | | | | | | | Total score^b^ |
| --- | --- | --- | --- | --- | --- | --- | --- | --- | --- | --- |
|  | Q1 | Q2 | Q3 | Q4 | Q5 | Q6 | Q7 | Q8 | Q9 |  |
| Morichi et al. [31] (2018) | Y | Y | Y | Y | Y | Y | U | N | Y | 7/9 |
| Fedecostante et al. [37] (2024) | Y | Y | Y | Y | Y | Y | U | N | Y | 7/9 |
| Morandi et al. [33] (2020) | Y | Y | Y | N | Y | Y | U | N | Y | 6/9 |
| Sabbe et al. [34] (2021) | Y | Y | Y | Y | Y | Y | Y | N | Y | 8/9 |
| McCusker et al. [32] (2011) | Y | Y | Y | Y | Y | Y | U | N | Y | 7/9 |
| Teale et al. [36] (2017) | Y | Y | Y | Y | U | Y | Y | N | Y | 7/9 |
| Sandberg et al. [35] (1998) | Y | Y | N | Y | Y | Y | Y | N | Y | 7/9 |
| Laurila et al. [28] (2003)^c^ | Y | Y | Y | Y | Y | Y | U | N | Y | 7/9 |
| Pitkala et al. [29] (2005)^c^ | Y | Y | N | N | Y | Y | U | N | Y | 5/9 |

^a^ Q1 - Q9 refer to questions 1-9 of the Joanna Briggs Institute (JBI) Critical Appraisal Checklist for Studies Reporting Prevalence Data [21] (**Supplementary Material 2**). Y (Yes) = 1 point, U (Unclear) = 0 points, N (No) = 0 points.

^b^ Higher scores indicate a lower risk of bias.

^c^ Both studies [28, 29] reported results based on the same sample and data collection process.

**Supplementary Material 4: Quality assessment of all included studies reporting associated factors**

| **Source (year)** | **Criteria and corresponding ratings^a^** | | | | | | | | **Total score^b^** |
| --- | --- | --- | --- | --- | --- | --- | --- | --- | --- |
|  | Q1 | Q2 | Q3 | Q4 | Q5 | Q6 | Q7 | Q8 |  |
| **Morichi et al. [31]** (2018) | Y | Y | Y | N/A | Y | Y | U | Y | 6/7 |
| **Fedecostante et al. [37]** (2024) | Y | Y | U | N/A | Y | Y | U | Y | 5/7 |
| **Sabbe et al. [34]** (2021) | Y | Y | Y | N/A | N | Y | Y | N | 5/7 |
| **McCusker et al. [32]** (2011) | Y | Y | Y | N/A | N | Y | U | U | 4/7 |

^a^ Q1-Q8 refer to questions 1-8 of the Joanna Briggs Institute (JBI) Critical Appraisal Checklist for Analytical Cross Sectional Studies [25] (**Supplementary Material 2**). Y (Yes) = 1 point, U (Unclear) = 0 points, N (No) = 0 points, N/A (Not applicable).

^b^ Higher scores indicate a lower risk of bias.

**Supplementary Material 5: PRISMA 2020 Checklist**

| **Section and Topic** | **Item #** | **Checklist item** | **Location where item is reported** |
| --- | --- | --- | --- |
| **TITLE** | | |  |
| Title | 1 | Identify the report as a systematic review. | Title page |
| **ABSTRACT** | | |  |
| Abstract | 2 | See the PRISMA 2020 for Abstracts checklist. | - |
| **INTRODUCTION** | | |  |
| Rationale | 3 | Describe the rationale for the review in the context of existing knowledge. | Page 2 |
| Objectives | 4 | Provide an explicit statement of the objective(s) or question(s) the review addresses. | Page 2 |
| **METHODS** | | |  |
| Eligibility criteria | 5 | Specify the inclusion and exclusion criteria for the review and how studies were grouped for the syntheses. | Page 3-4 |
| Information sources | 6 | Specify all databases, registers, websites, organisations, reference lists and other sources searched or consulted to identify studies. Specify the date when each source was last searched or consulted. | Page 3 |
| Search strategy | 7 | Present the full search strategies for all databases, registers and websites, including any filters and limits used. | Supplementary Material 1 |
| Selection process | 8 | Specify the methods used to decide whether a study met the inclusion criteria of the review, including how many reviewers screened each record and each report retrieved, whether they worked independently, and if applicable, details of automation tools used in the process. | Page 4 |
| Data collection process | 9 | Specify the methods used to collect data from reports, including how many reviewers collected data from each report, whether they worked independently, any processes for obtaining or confirming data from study investigators, and if applicable, details of automation tools used in the process. | Page 4 |
| Data items | 10a | List and define all outcomes for which data were sought. Specify whether all results that were compatible with each outcome domain in each study were sought (e.g. for all measures, time points, analyses), and if not, the methods used to decide which results to collect. | Page 4, see tables 1, 2 & 3 |
|  | 10b | List and define all other variables for which data were sought (e.g. participant and intervention characteristics, funding sources). Describe any assumptions made about any missing or unclear information. | Page 4, see tables 1, 2 & 3 |
| Study risk of bias assessment | 11 | Specify the methods used to assess risk of bias in the included studies, including details of the tool(s) used, how many reviewers assessed each study and whether they worked independently, and if applicable, details of automation tools used in the process. | Page 4 |
| Effect measures | 12 | Specify for each outcome the effect measure(s) (e.g. risk ratio, mean difference) used in the synthesis or presentation of results. | Page 4 |
| Synthesis methods | 13a | Describe the processes used to decide which studies were eligible for each synthesis (e.g. tabulating the study intervention characteristics and comparing against the planned groups for each synthesis (item #5)). | Page 4 |
|  | 13b | Describe any methods required to prepare the data for presentation or synthesis, such as handling of missing summary statistics, or data conversions. | Page 4 |
|  | 13c | Describe any methods used to tabulate or visually display results of individual studies and syntheses. | Page 4 |
|  | 13d | Describe any methods used to synthesize results and provide a rationale for the choice(s). If meta-analysis was performed, describe the model(s), method(s) to identify the presence and extent of statistical heterogeneity, and software package(s) used. | Page 4 |
|  | 13e | Describe any methods used to explore possible causes of heterogeneity among study results (e.g. subgroup analysis, meta-regression). | Page 4 |
|  | 13f | Describe any sensitivity analyses conducted to assess robustness of the synthesized results. | Page 4 |
| Reporting bias assessment | 14 | Describe any methods used to assess risk of bias due to missing results in a synthesis (arising from reporting biases). | Page 4 |
| Certainty assessment | 15 | Describe any methods used to assess certainty (or confidence) in the body of evidence for an outcome. | - |
| **RESULTS** | | |  |
| Study selection | 16a | Describe the results of the search and selection process, from the number of records identified in the search to the number of studies included in the review, ideally using a flow diagram. | Page 5, see figure 1 |
|  | 16b | Cite studies that might appear to meet the inclusion criteria, but which were excluded, and explain why they were excluded. | Page 5 |
| Study characteristics | 17 | Cite each included study and present its characteristics. | See tables 1 & 2 |
| Risk of bias in studies | 18 | Present assessments of risk of bias for each included study. | See tables 2 & 3, Supplementary Material 3 & 4 |
| Results of individual studies | 19 | For all outcomes, present, for each study: (a) summary statistics for each group (where appropriate) and (b) an effect estimate and its precision (e.g. confidence/credible interval), ideally using structured tables or plots. | See tables 1, 2 & 3 |
| Results of syntheses | 20a | For each synthesis, briefly summarise the characteristics and risk of bias among contributing studies. | Page 5 & 9 |
|  | 20b | Present results of all statistical syntheses conducted. If meta-analysis was done, present for each the summary estimate and its precision (e.g. confidence/credible interval) and measures of statistical heterogeneity. If comparing groups, describe the direction of the effect. | Page 9, see figure 2, 3 & 4 |
|  | 20c | Present results of all investigations of possible causes of heterogeneity among study results. | Page 9, see figure 2 & 3 |
|  | 20d | Present results of all sensitivity analyses conducted to assess the robustness of the synthesized results. | Page 9, see figure 3 |
| Reporting biases | 21 | Present assessments of risk of bias due to missing results (arising from reporting biases) for each synthesis assessed. | Page 9 & 14, see tables 1, 2 & 3, figure 4 |
| Certainty of evidence | 22 | Present assessments of certainty (or confidence) in the body of evidence for each outcome assessed. | Page 15-17 |
| **DISCUSSION** | | |  |
| Discussion | 23a | Provide a general interpretation of the results in the context of other evidence. | Page 14-17 |
|  | 23b | Discuss any limitations of the evidence included in the review. | Page 14-17 |
|  | 23c | Discuss any limitations of the review processes used. | Page 17 |
|  | 23d | Discuss implications of the results for practice, policy, and future research. | Page 15-17 |
| **OTHER INFORMATION** | | |  |
| Registration and protocol | 24a | Provide registration information for the review, including register name and registration number, or state that the review was not registered. | Page 3 |
|  | 24b | Indicate where the review protocol can be accessed, or state that a protocol was not prepared. | Page 3 |
|  | 24c | Describe and explain any amendments to information provided at registration or in the protocol. | - |
| Support | 25 | Describe sources of financial or non-financial support for the review, and the role of the funders or sponsors in the review. | Page 18 |
| Competing interests | 26 | Declare any competing interests of review authors. | Page 18 |
| Availability of data, code and other materials | 27 | Report which of the following are publicly available and where they can be found: template data collection forms; data extracted from included studies; data used for all analyses; analytic code; any other materials used in the review. | - |

From: Page MJ, McKenzie JE, Bossuyt PM, Boutron I, Hoffmann TC, Mulrow CD, et al. The PRISMA 2020 statement: an updated guideline for reporting systematic reviews. BMJ 2021;372:n71. doi: 10.1136/bmj.n71. This work is licensed under CC BY 4.0. To view a copy of this license, visit https://creativecommons.org/licenses/by/4.0/
